# Transcutaneous Auricular Vagus Nerve Stimulation for Chronic Insomnia Disorder: A Randomized Clinical Trial

**DOI:** 10.1001/jamanetworkopen.2024.51217

**Published:** 2024-12-16

**Authors:** Shuai Zhang, Yanan Zhao, Zongshi Qin, Ying Han, Jiakai He, Bin Zhao, Lei Wang, Yuting Duan, Jin Huo, Tuoran Wang, Yu Wang, Peijing Rong

**Affiliations:** 1 Institute of Acupuncture and Moxibustion, China Academy of Chinese Medical Sciences, Beijing, China; 2 Department of Geriatrics, Dongzhimen Hospital, Beijing University of Chinese Medicine, Beijing, China; 3 Clinical Research Institute, Institute of Advanced Clinical Medicine, Peking University, Beijing, China; 4 Acupuncture and Moxibustion Hospital, China Academy of Chinese Medical Sciences, Beijing, China; 5 Department of Traditional Chinese Medicine, Peking University People’s Hospital, Beijing, China; 6 Department of Acupuncture, College of traditional Chinese medicine, Southern Medical University, Guangzhou, China; 7 Institute of Basic Research in Clinical Medicine, China Academy of Chinese Medical Sciences, Beijing, China; 8 The Affiliated Traditional Chinese Medicine Hospital, Guangzhou Medical University, Guangzhou, China

## Abstract

**IMPORTANCE:**

Evidence from randomized clinical trials of transcutaneous auricular vagus nerve stimulation (taVNS) for chronic insomnia disorder is lacking.

**OBJECTIVE:**

To evaluate the efficacy and safety of taVNS for chronic insomnia compared with the sham taVNS.

**DESIGN, SETTING, and PARTICIPANTS:**

This randomized clinical trial was conducted from October 2021 to December 2022 at a single center in Beijing, China. Patients with chronic insomnia disorder with a Pittsburgh Sleep Quality Index (PSQI) of at least 8 were enrolled. Statistical analysis was performed from June to September 2023.

**INTERVENTIONS:**

Patients were allocated to the active taVNS group or sham taVNS group with a 1:1 ratio. Both groups received the stimulation for 30 minutes each time, twice a day, 5 consecutive days a week, with an 8-week treatment and a 12-week follow-up.

**MAIN OUTCOMES AND MEASURES:**

The primary end point was the mean change from baseline through week 8 in PSQI scores. Minimal clinically important difference was 2.5 points. Secondary outcomes included mental health, sleepiness, and fatigue. Safety was also evaluated.

**RESULTS:**

A total of 72 participants were randomized to either active taVNS group (36 participants; mean [SD] age, 45.2 [14.5] years; 27 [75.0%] female) or the sham taVNS group (36 participants; mean [SD] age, 44.6 [13.9] years; 31 [86.1%] female); 68 participants completed the 8-week intervention. The least-square mean changes from baseline to week 8 in PSQI were −8.2 (95% CI, −9.3 to −7.0) points in the taVNS group and −3.9 (95% CI, −5.1 to −2.7) points in the sham group. Both groups experienced statistically significant improvements from before to after the intervention. However, active taVNS showed a clinically meaningful 4.2-point greater reduction (95% CI, −5.9 to −2.6 points; *P* < .001; Cohen *d* effect size, 1.2) in PSQI compared with the sham group (minimal clinically important difference = 2.5 points). Secondary outcomes, including mental health and fatigue, showed similar favorable results. The efficacy of taVNS was sustained throughout the 20-week study period.

**CONCLUSIONS AND RELEVANCE:**

In this randomized clinical trial, taVNS significantly reduced insomnia severity. Clinically meaningful enhancements in PSQI scores were observed compared with sham stimulation, with the benefits of taVNS sustained over a 20-week period. Future multicenter clinical trials with large sample sizes are needed to validate its effectiveness across diverse populations.

**Trial Registration:**

Chinese Clinical Trial Registry: ChiCTR2100051319

## Key Points

**Question:**

What is the safety and efficacy profile of transcutaneous auricular vagus nerve stimulation (taVNS) in patients with chronic insomnia disorder?

**Findings:**

This parallel-group, sham-controlled randomized clinical trial of 72 patients with chronic insomnia disorder found that taVNS achieved clinically significant improvements in patients with chronic insomnia disorder compared with sham stimulation.

**Meaning:**

These results suggest that taVNS would be safe and effective in treating chronic insomnia.

## Teaser

This randomized clinical trial investigates the efficacy and safety of transcutaneous auricular vagus nerve stimulation (taVNS) for chronic insomnia compared with sham taVNS among patients in China.

## Introduction

Insomnia is a major health care problem, defined as a sleep continuity disorder with various daytime complaints, including excessive sleepiness, fatigue, mood and cognitive disturbances, as well as concerns and dissatisfaction with sleep,^1^ which affects approximately one-third of the global adult population.^2^ Currently, the primary therapeutic modalities of insomnia are cognitive-behavioral therapy for insomnia (CBT-I) and medications.^3,4,5^ However, face-to-face training is required for CBT-I, and long-term medication use increases side effects, including daytime drowsiness, dizziness, memory problems, and impaired coordination.^6^ There remains a need for the development of new treatment approaches.

Transcutaneous auricular vagus nerve stimulation (taVNS), a nonpharmacological intervention, has shown efficacy in treating conditions such as depression,^7,8^ epilepsy,^9,10^ and migraine,^11^ and it has gained attention in the treatment of insomnia in recent years.^12,13,14,15^ Previous studies have demonstrated that taVNS cannot only effectively improve sleep quality in patients with insomnia, but also alleviate accompanying symptoms such as anxiety, depression, daytime sleepiness, and fatigue.^12,13,16^

Although taVNS therapy has made some advances in the treatment of insomnia, the factors influencing its clinical effectiveness remain unclear. Previous research has shown that mean Pittsburgh Sleep Quality Index (PSQI) scores in insomnia cases fluctuate between 8.8 to 11.6 after 4-week treatment,^12,15,16^ suggesting a need for substantial improvement in clinical symptoms, whereas other interventions such as electroacupuncture requires an optimal treatment course of 8 weeks to effectively address insomnia,^17^ and CBT-I for insomnia is 6 to 8 weeks.^18,19^ This prompts the hypothesis that extending the taVNS treatment course may enhance its clinical efficacy in treating insomnia. Therefore, this study extended 8-week taVNS therapy for chronic insomnia disorder. Furthermore, stimulation of the auricular points in the scapha (absence of vagus nerve distribution) was used as the sham taVNS group in previous research, and found that taVNS could significantly reduce the scores of PSQI, but there was no significant difference compared with the sham taVNS group.^12,15^ In addition, some scholars take electrical stimulation as an example to understand neural regulation technology from the perspective of energy, and think that the current intensity affects the energy given by electrical stimulation, that is, the greater the current given, the greater the energy.^20^ According to the output parameters of the SDZ-IIB Hwato electroacupuncture instrument, its minimum output current intensity (0.1 mA) was taken as the stimulation amount of the sham control group, and its energy was considered to be the minimum.

The present study, Transcutaneous Auricular Stimulation With Chronic Insomnia (TASC-I), was designed to evaluate the safety and effectiveness of taVNS in patients with chronic insomnia disorder. We hypothesized that taVNS would show greater improvement in sleep severity after 8 weeks of treatment. A rigorous randomized clinical trial with a sham control group was designed to address this aim in an extended spectrum of patients with chronic insomnia.

## Methods

### Study Design

This TASC-I trial was a randomized clinical trial designed by the investigators. Patients were randomly assigned 1:1 to receive taVNS and sham taVNS. Long-term efficacy was measured at 20 weeks. The trial was conducted at the Acupuncture and Moxibustion Hospital, China Academy of Chinese Medical Sciences (CACMS) from October 25, 2021, to December 8, 2022. The study protocol was approved by the Institute of Acupuncture and Moxibustion, CACMS institutional review board. All participants provided written informed consent. The trial was prospectively registered in the Chinese Clinical Trial Registry (ChiCTR2100051319). This study followed the Consolidated Standards of Reporting Trials (CONSORT) reporting guideline.

### Study Population and Recruitment

Qualified participants were recruited via hospital outpatient clinics, hospital WeChat official accounts, and posters at Acupuncture and Moxibustion Hospital, CACMS. Physicians at the Department of Encephalopathy conducted evaluations and screenings. Inclusion criteria encompassed meeting the diagnostic criteria for insomnia disorder in the *Diagnostic and Statistical Manual of Mental Disorders* (Fifth Edition)^21^ experiencing insomnia for at least 3 months, at least 3 nights a week, and being aged between 18 to 70 years. Patients were required to report a total score of at least 8 on the Pittsburgh Sleep Quality Index (PSQI) and abstain from hypnotics for at least 1 month before treatment. Participants with snoring, sleep apnea, narcolepsy, and restless leg syndrome were excluded through symptom screening, as well as patients with serious psychosomatic and organic diseases. Those with circadian rhythm disorders or receiving long-term hypnotics were also excluded. Patients were not compensated for study participation. A complete list of eligibility criteria appears in Supplement 1.

### Randomization and Blinding

Eligible patients were randomly assigned in a 1:1 ratio to either the taVNS group or the sham taVNS group. Randomization was performed using a computer-generated random numbers table with a block size of 4. Numbers were allocated sequentially and sealed in an opaque envelope by a research assistant. Patients, outcome assessors, and statisticians were blinded to treatment assignment. The blinding for participants were assessed by the Bang blinding index (BBI); when the BBI value ranges from −0.2 to 0.2, blinding is considered to be successful.

### Intervention and Control

The intervention protocol was based on a preliminary clinical trial,^12^ which utilized an electroacupuncture instrument (Hwato, SDZ-II B, Suzhou, China) and special silica gel ear clips. Patients in both groups received electrical stimulation at the bilateral auricular cymba conchae (auricular point kidney, CO_10_) and cavum conchae (auricular point heart, CO_15_), where there is rich vagus nerve distribution.^22^ The illustration of taVNS procedure was described in eFigure 1 in Supplement 2. Patients in taVNS group received stimulation parameters, including dilatational wave at 4/20 Hz (4 Hz for 5 seconds, 20 Hz for 10 seconds, alternately), pulse width of 0.2 ms ±30%, and intensity adjusted to the patient’s maximum tolerable level (0.8-1.5 mA). The sham taVNS group received the same parameters with a current intensity of 0.1 mA. All participants underwent the intervention for 30 minutes, twice a day, 5 consecutive days a week, for 8 weeks, followed by a 12-week follow-up period. After screening, all patients were trained to apply taVNS or sham taVNS. All treatments were self-administered by patients or with the assistance of family members at home. Patients were also instructed to complete a sleep treatment diary each day to write the exact treatment time, date, sleep situation, and describe any side effects corresponding with or temporarily related to treatment. The investigators checked all diaries every 2 weeks via WeChat (an instant messaging app, allowing its users to send words, emojis, pictures, calls, and video calls one-on-one with contacts) video to enhance compliance.

### Outcome Measurements

The primary outcome was the change in PSQI from baseline to week 8.^23^ PSQI is a widely used questionnaire to assess sleep quality for the past month, with 7 components for the features of sleep. A change score of 4 points (vs baseline) in the PSQI is considered to be the minimal important change (MIC), and a difference score of 2.5 points (vs control group) is considered to be the minimal clinically important difference (MCID).^24,25^ Secondary outcomes included comparisons of the taVNS and sham taVNS groups at week 4, 8, and 20 of Insomnia Severity Index (ISI),^26^ Hamilton Depression Scale (HAMD; assessing the degree of depression in patients with insomnia),^27^ Hamilton Anxiety Scale (HAMA; assessing the anxiety level of patients with insomnia),^28^ Epworth Sleepiness Scale (ESS; assessing daytime sleepiness in patients with insomnia),^29^ and Flinders Fatigue Scale (FFS; assessing the degree of daytime fatigue in patients with insomnia).^30^ The response analysis was conducted at 4, 8, and 20 weeks. In this study, the responder was defined as the percentage of participants with a reduction of at least 50% from baseline in the PSQI score after treatment. All continuous outcomes were evaluated with Cohen *d* effect size as point estimates with 95% CI. Adverse reactions were reported by patients and recorded by the physicians.

### Sample Size Calculations

Estimation of sample size was based on the results of the preliminary study.^16^ At the end of 8 weeks of treatment, the mean PSQI score for sham taVNS group is 14.9, and 11.1 for taVNS group. To detect a difference of 3.8 between groups in PSQI score, assuming an SD of 4.3 and 2-sided significance level of 5%. Considering the dropout and loss to follow-up rate of 25%, and with 0.05 α-level and 90% power, the total sample size required for the trial was 72 patients.

### Statistical Analysis

Baseline demographics were reported descriptively using the intent-to-treat (ITT) population. All efficacy analyses were performed using the ITT set and per-protocol (PP) set, defined as all randomized participants who received at least 6 weeks of intervention. The difference in change from baseline to week 8 in PSQI total score was estimated using a mixed model for repeated measures. The model included terms for intervention (taVNS or sham taVNS), visit (weeks 4, 8, 20), intervention by visit interaction, and baseline PSQI score. Least-squares mean change from baseline and least-squares mean difference between the groups at week 8 along with 95% CI and a 2-sided *P* value were estimated for the primary end point. Continuous secondary outcomes were analyzed in the same methods as the primary outcome analysis. Cohen *d* effect size was calculated using the absolute value of the least-squares mean difference between groups in score from baseline at week 8 divided by the pooled SD estimated from the mixed model for repeated measures. The percentage of PSQI responders at weeks 4, 8, and 20 were calculated and summarized by treatment group. The percentage of PSQI responders was compared between groups using the χ^2^ test. Adverse events (AEs) were recorded via participant self-reports and physician assessments. At each study visit, participants were asked about any new symptoms or health issues. AEs were classified by severity and assessed for their relationship to the study treatment. The Fisher exact test was used to compare the incidence of AEs between the groups. Statistical analysis was conducted from June to September 2023 using SAS version 9.4 (SAS Institute), with significance set at 2-sided *P* < .05.

## Results

**Figure 1.**
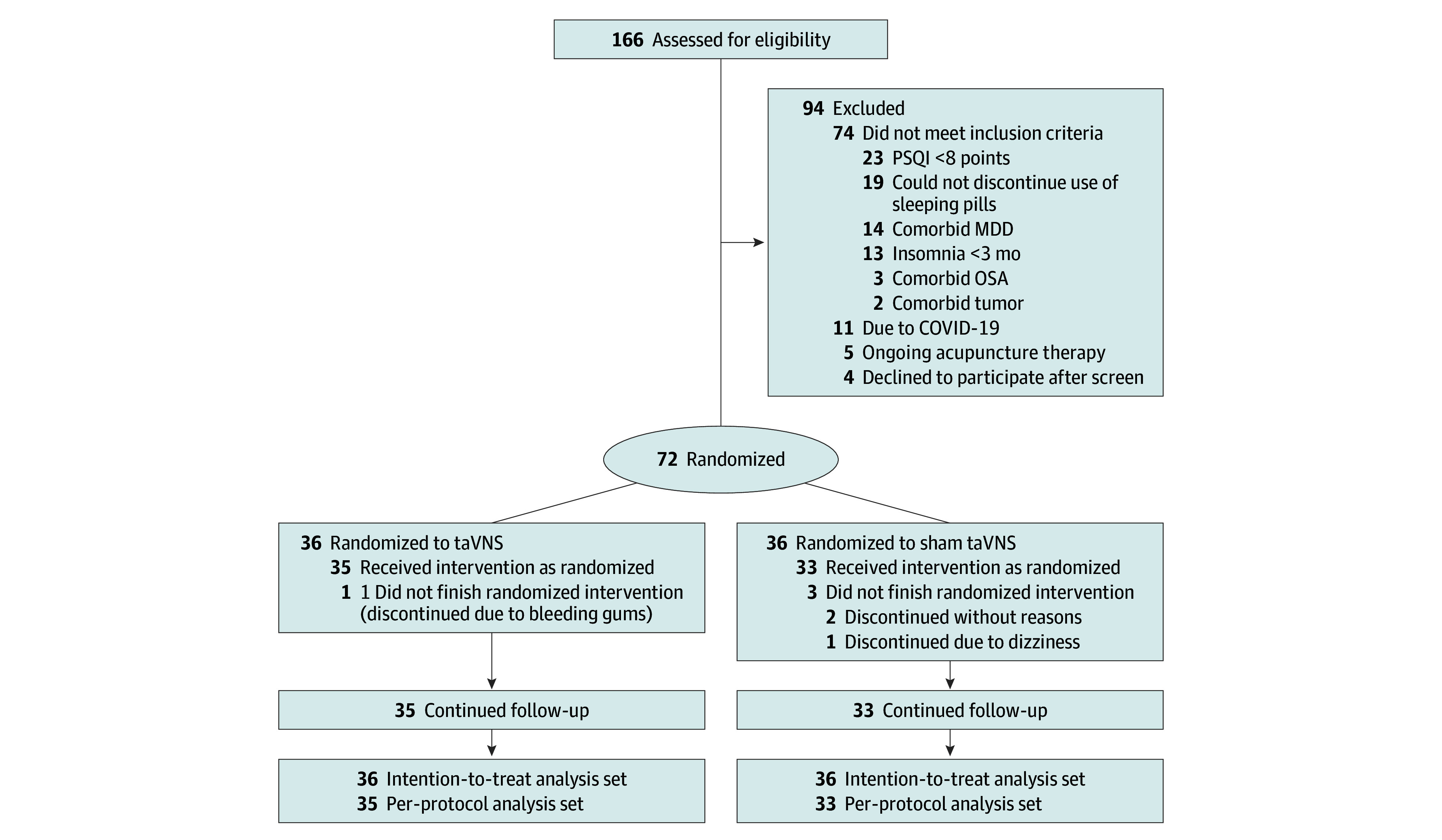
Study Flowchart MDD, major depressive disorder; OSA, obstructive sleep apnea; PSQI, Pittsburgh Sleep Quality Index; taVNS, transcutaneous auricular vagus nerve stimulation.

Between October 2021 and December 2022, 166 patients were screened for eligibility, of whom 23 had PSQI of 8 or lower, 19 could not discontinue the use of sleeping pills, 14 had comorbid major depressive disorder, 13 had insomnia less than 3 months, 3 had comorbid obstructive sleep apnea, 2 had a comorbid tumor, 4 failed to attend after enrollment, 5 had ongoing acupuncture treatment, and 11 dropped out due to the impact of the COVID-19 pandemic. A total of 72 patients, 36 in the taVNS group (mean [SD] age, 45.2 [14.5] years; 27 [75.0%] female) and 36 in the sham taVNS group (mean [SD] age, 44.6 [13.9] years; 31 [86.1%] female), were randomly assigned; 2 patients did not complete treatment as required, 1 in each group. In the sham taVNS group, 1 dropped out due to dizziness during the second week after intervention and 2 were lost at 4 and 8 weeks without reason. In the taVNS group, 1 dropped out due to bleeding group during the first week after intervention. Thus, 68 (94.4%) completed the 8-week treatment and finished the follow-up period. At baseline, there was no difference in all variables between groups. All participants responded to the blinding assessment questionnaire. The result of the blinding assessment showed that 15 patients (25%) in the sham taVNS group guessed incorrectly about their group assignment (Bang blinding index [BBI], −0.19 [95% CI, −0.43 to 0.04]), whereas 8 (22.9%) in the taVNS group also guessed incorrectly (BBI, 0.31 [95% CI, 0.07 to 0.54]). This indicated successful blinding for the sham taVNS group (eFigure 2 and eTable 1 in Supplement 2). The participant recruitment flowchart is shown in Figure 1. Baseline demographics and characteristics are summarized in Table 1. It was found that the mean treatment times of taVNS group and sham taVNS group were 76 and 72 respectively, both greater than 80% of the treatment times.

**Table 1.** Baseline Demographics and Characteristics

Characteristics	Participants, No. (%)	
	taVNS (n = 36)	Sham taVNS (n = 36)
Mean age (SD), y	45.2 (14.5)	44.6 (13.9)
Age, y		
≤40	18 (50.0)	15 (41.7)
41-59	10 (27.8)	15 (41.7)
60-70	8 (22.2)	6 (16.6)
Sex		
Male	9 (25.0)	5 (13.9)
Female	27 (75.0)	31 (86.1)
BMI, mean (SD)	25.8 (4.5)	25.5 (4.6)
Duration, median (IQR), mo	60 (84)	60 (96)
Education		
High school education or below	6 (16.7)	9 (25)
University education	16 (44.4)	16 (44.4)
Graduate school or above	14 (38.9)	11(30.6)
Marriage status		
Married	28 (77.8)	23 (63.9)
Single	8 (22.2)	13 (36.1)
Tea or coffee consumption, times/d		
Never	18 (50.0)	16 (44.4)
<1	11 (30.8)	14 (38.9)
1	4 (11.1)	5 (13.9)
≥2	3 (8.3)	1 (2.7)
Previous use of sleeping pills	6 (16.7)	7 (19.4)
Family history of insomnia	10 (27.8)	9 (25.0)
Combined diseases	25 (69.4)	21 (58.3)
Baseline PSQI score, mean (SD)	13.8 (2.6)	13.3 (2.4)
Baseline ISI score, mean (SD)	16.4 (4.1)	15.9 (3.2)
Baseline HAMD score, mean (SD)	8.4 (3.1)	7.9 (2.7)
Baseline HAMA score, mean (SD)	9.0 (2.7)	7.7 (3.1)
Baseline ESS score, mean (SD)	7.1 (4.9)	7.8 (5.2)
Baseline FFS score, mean (SD)	12.5 (6.9)	10.4 (6.5)

Abbreviations: BMI, body mass index (calculated as weight in kilograms divided by height in meters squared); ESS, Epworth Sleepiness Scale; FFS, Flinders Fatigue Scale; HAMA, 14-item Hamilton Anxiety; HAMD, 17-item Hamilton Depression; ISI, Insomnia Severity Index; PSQI, Pittsburgh Sleep Quality Index; taVNS, transcutaneous auricular vagus nerve stimulation.

### Primary Efficacy End Point

**Figure 2.**
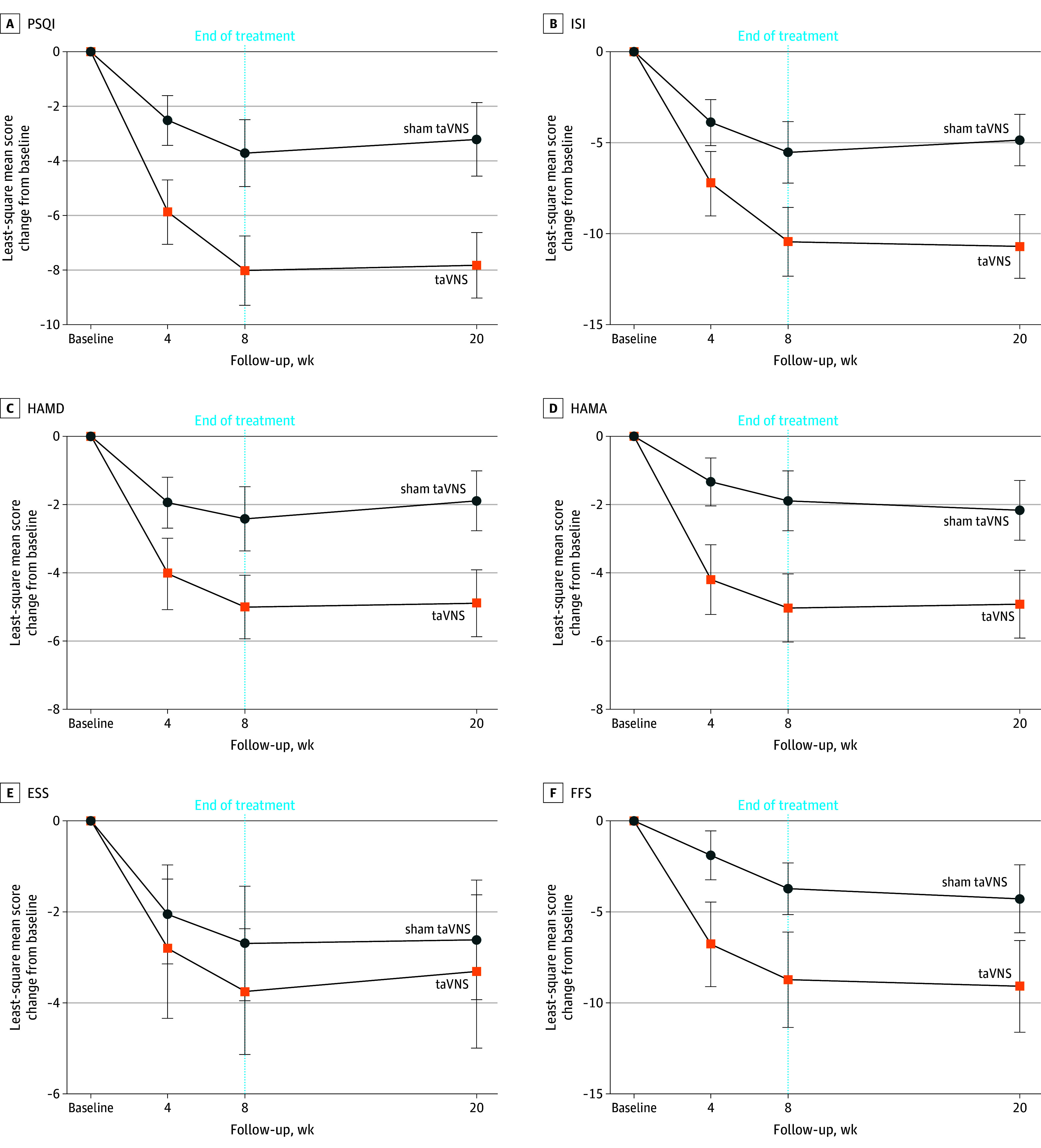
Primary and Secondary Outcomes at the Middle of Treatment (Week 4), the End of Treatment (Week 8), and the End of Follow-Up (Week 20) Comparison between the 2 groups in the mean change of PSQI (A), ISI (B), HAMD (C), HAMA (D), ESS (E), and FFS (F) score at weeks 4, 8, and 20. The point estimates and interval estimates represented as least-square mean change and 95% CIs, respectively. ESS indicates Epworth Sleepiness Scale; FFS, Flinders Fatigue Scale; HAMA, Hamilton Anxiety Scale; HAMD, Hamilton Depression Scale; ISI, Insomnia Severity Index; PSQI, Pittsburgh Sleep Quality Index; taVNS, transcutaneous auricular vagus nerve stimulation.

All participants (36 per group) were included in the ITT population for the primary end point analysis. For the primary end point, the least-squares mean change from baseline to week 8 in PSQI was −8.2 (95% CI, −9.3 to −7.0) compared with −3.9 (95% CI, −5.1 to −2.7) in the sham group. At week 8, the taVNS group exhibited significantly greater, clinically meaningful reductions in PSQI total score than the sham taVNS group (compared with baseline, MIC = 4), and PSQI was meaningfully and significantly lower in the taVNS compared with sham taVNS group (least-squares mean difference, −4.2 [95% CI, −5.9 to −2.6]; *P* < .001; Cohen *d* effect size, 1.2; MCID = 2.5; (Table 2). Among participants receiving active taVNS, a statistically significant improvement in PSQI vs sham taVNS was evident starting at week 4 (mean difference, −3.4 [95% CI, −4.7 to −2.1]; *P* < .001; Cohen *d* effect size, 1.1) and was enhanced through the end of the trial at week 20 (mean difference, −4.5 [95% CI, −6.4 to −2.6]; *P* < .001; Cohen *d* effect size, 1.2) (Figure 2A). A supportive analysis of change from baseline to week 8 in PSQI score for PP set supported the primary analysis (eTable 2 in Supplement 2).

**Table 2.** Comparison of Primary and Secondary Outcomes Between the 2 Groups

Outcomes	taVNS (n = 36)	Sham taVNS (n = 36)	*P* value	Difference (95%CI)	Cohen *d* (95%CI)
**Least-square mean changes from baseline in PSQI score (95% CI)**					
Week 4	−6.0 (−6.9 to −5.1)	−2.6 (−3.5 to −1.7)	<.001	−3.4 (−4.7 to −2.1)	1.1 (0.6 to 1.6)
Week 8	−8.2 (−9.3 to −7.0)	−3.9 (−5.1 to −2.7)	<.001	−4.2 (−5.9 to −2.6)	1.2 (0.7 to 1.7)
Week 20	−8.0 (−9.3 to −6.6)	−3.4 (−4.8 to −2.0)	<.001	−4.5 (−6.4 to −2.6)	1.2 (0.7 to 1.7)
**Responders, % (95% CI)**					
Week 4	50.0 (33.2 to 66.8)	16.7 (7.0 to 33.5)	.003	33.3 (9.6 to 52.7)	NA
Week 8	69.4 (51.7 to 83.1)	27.8 (14.2 to 45.2)	<.001	41.6 (20.7 to 62.7)	NA
Week 20	72.2 (54.6 to 85.2)	22.2 (10.7 to 39.6)	<.001	50.0 (25.2 to 67.3)	NA
**Least-square mean changes from baseline in ISI score (95% CI)**					
Week 4	−7.4 (−8.7 to −6.0)	−4.0 (−5.3 to −2.7)	<.001	−3.4 (−5.2 to −1.5)	0.7 (0.3 to 1.2)
Week 8	−10.6 (−12.3 to −8.9)	−5.9 (−7.6 to −4.2)	<.001	−4.7 (−7.1 to −2.3)	0.9 (0.4 to 1.4)
Week 20	−10.8 (−12.7 to −9.0)	−5.2 (−7.1 to −3.3)	<.001	−5.6 (−8.3 to −3.0)	1.2 (0.7 to 1.8)
**Least-square mean changes from baseline in HAMD score (95% CI)**					
Week 4	−4.1 (−5.0 to −3.4)	−2.0 (−2.8 to −1.2)	<.001	−2.1 (−3.2 to −1.0)	0.8 (0.3 to 1.3)
Week 8	−5.2 (−6.1 to −4.2)	−2.6 (−3.6 to −1.6)	<.001	−2.6 (−4.0 to −1.2)	0.9 (0.4 to 1.4)
Week 20	−5.0 (−6.2 to −3.9)	−2.0 (−3.1 to −0.9)	<.001	−3.0 (−4.6 to −1.5)	1.1 (0.6 to 1.6)
**Least-square mean changes from baseline in HAMA score (95% CI)**					
Week 4	−4.3 (−5.0 to −3.6)	−1.4 (−2.1 to −0.7)	<.001	−2.9 (−4.0 to −1.9)	1.1 (0.6 to 1.6)
Week 8	−5.2 (−6.1 to −4.2)	−2.0 (−2.9 to −1.1)	<.001	−3.2 (−4.5 to −1.8)	1.1 (0.6 to 1.6)
Week 20	−5.1 (−6.1 to −4.0)	−2.3 (−3.4 to −1.2)	<.001	−2.8 (−4.3 to −1.2)	1.0 (0.5 to 1.5)
**Least-square mean changes from baseline in ESS score (95% CI)**					
Week 4	−2.9 (−4.0 to −1.8)	−2.2 (−3.3 to −1.1)	.34	−0.8 (−2.3 to 0.8)	0.2 (−0.3 to 0.7)
Week 8	−3.9 (−5.3 to −2.5)	−2.7 (−3.9 to −1.4)	.35	−1.0 (−3.0 to 1.0)	0.3 (−0.2 to 0.7)
Week 20	−3.5 (−5.1 to −1.9)	−2.6 (−3.9 to −1.3)	.64	−0.5 (−2.8 to 1.7)	0.2 (−0.2 to 0.7)
**Least-square mean changes from baseline in FFS score (95% CI)**					
Week 4	−6.9 (−8.5 to −5.4)	−1.8 (−3.4 to −0.3)	<.001	−5.1 (−7.3 to −2.9)	0.4 (−0.1 to 0.9)
Week 8	−8.9 (−11.0 to −6.9)	−3.9 (−6.0 to −1.9)	.001	−5.0 (−7.8 to −2.1)	0.4 (−0.1 to 0.8)
Week 20	−9.3 (−11.5 to −7.0)	−4.6 (−6.9 to −2.3)	.005	−4.7 (−7.9 to −1.4)	0.4 (−0.1 to 0.9)

Abbreviations: ESS, Epworth Sleepiness Scale; FFS, Flinders Fatigue Scale; HAMA, 14-item Hamilton Anxiety; HAMD, 17-item Hamilton Depression; ISI, Insomnia Severity Index; PSQI, Pittsburgh Sleep Quality Index; taVNS, Transcutaneous auricular vagus nerve stimulation.

### Secondary Efficacy Outcomes Measures

More patients in the taVNS group reported marked or moderate symptom improvements at each assessment point. The percentage of PSQI responders (at least 50% improvement from baseline) at week 4, 8, and 20 was 50.0%, 69.4%, and 72.2% in taVNS group, compared with 16.7%, 27.8%, and 22.2% in the sham group (ratio difference: 33.3%, 41.6%, and 50% at week 4, 8, and 20) (Table 2). Among the 7 components of PSQI, the taVNS group had a higher reduction in subjective sleep quality, sleep latency, sleep duration, habitual sleep efficiency and daytime dysfunction scores than the sham taVNS group, and no differences in sleep disturbances and use of hypnotic drugs scores (eFigure 3 and eTable 3 in Supplement 2). The results of ISI showed similar patterns as PSQI, the least-squares mean change from baseline in the ISI score in taVNS group was significantly higher than the sham taVNS group with between-group differences of −3.4 (95% CI, −5.2 to −1.5; *P* < .001; Cohen *d* effect size, 0.7) at week 4, −4.7 (95% CI, −7.1 to −2.3; *P* < .001; Cohen *d* effect size, 0.9) at week 8, and −5.6 (95% CI, −8.3 to −3.0; *P* < .001; Cohen *d* effect size, 1.2) at week 20 (Figure 2B; Table 2). The taVNS group had greater decreases in HAMD, HAMA, and FFS scores. No significant differences were observed in ESS scores (Figure 2C-F; Table 2). A supportive analysis of change from baseline to week 8 in all outcomes for the PP analysis set showed the similar conclusion (eFigure 4 and eTable 2 in Supplement 3).

### Safety

Adverse events occurred in 5 patients (13.9%) in the taVNS group and 4 patients (11.1%) in the sham taVNS group. No significant difference was found among groups for the proportion of patients with AEs. The most commonly reported taVNS-related AEs were slight clamping pain in the outer ear (4 in taVNS group vs 3 in sham taVNS group) during the treatment, which disappeared by local pressing and kneading after the treatment. During treatment, bleeding gums occurred in 1 case (2.8%) in the taVNS group and vertigo occurred in 1 case (2.8%) in the sham taVNS group, both of which returned to normal 2 to 3 days after withdrawal from treatment. No patients had severe AEs in the trial (eTable 4 in Supplement 2).

## Discussion

This randomized clinical trial found that taVNS administered at the 0.8 to 1.5 mA intensity for 8 weeks effectively improved sleep quality in patients with chronic insomnia disorder, with sustained benefits lasting for 20 weeks after the end of treatment. taVNS was associated with a statistically significant and clinically meaningful 4.3-point greater reduction in PSQI score compared with the sham taVNS at week 8. A few previous clinical studies have used the maximum tolerable intensity to demonstrate the effects of taVNS treatment for 4 weeks on various aspects such as sleep quality, anxiety, depression, daytime fatigue, and sleepiness in patients with insomnia.^12,13,14^ There are a limited number of studies with an extended treatment course. To our knowledge, this is the first study investigating the effects of taVNS on treatment course of 8 weeks provided the dose-response for optimizing taVNS protocols for chronic insomnia disorder.

Compared with sham taVNS group, 8 weeks taVNS treatment significantly improved sleep quality, reduced insomnia severity, and alleviated accompanying symptoms of anxiety, depression, and fatigue, but not in daytime sleepiness. The differences were comparable from week 4, indicating an early onset of efficacy with taVNS group. The degree of daytime sleepiness in the 2 groups was not serious, which may be the reason why there was no significant difference between the 2 groups after treatment. In addition, this study found that the taVNS group demonstrated superiority over sham taVNS group in enhancing sleep quality, increasing total sleep time, enhancing sleep efficiency, shortening sleep latency, and improving the daytime function in patients with chronic insomnia disorder. In other words, taVNS can better improve the treatment response including shorten sleep latency, reduce awake time, prolong sleep time and improve sleep efficiency, which was similar to the efficacy of previous studies^12,14^ and the CBT-I.^31,32^

Studies have found that taVNS at 130 μA exhibits a more favorable potential for modulating cardiac autonomic function compared with taVNS at 90 μA.^33^ Furthermore, the taVNS with an intensity ranging from 2.0 to 2.2 mA can increase pupil diameter, which is significantly different from low- and high-intensity stimulation (0.5 mA or 3 mA).^34^ Studies on the effects of different intensities of respiratory-gated auricular vagal afferent nerve stimulation (RAVANS) on patients with hypertensive also found that mid-intensity RAVANS (mean [SD], 0.26 [0.15] mA) could significantly increase cardiovascular tone and reduce sympathetic tone during a paced breathing task, and help to reduce blood pressure compared with low-intensity (mean [SD], 0.10 [0.08] mA) and no stimulation.^35^ Studies on VNS found an inverted U-shaped relationship between the plasticity of motor cortex and auditory cortex and the stimulation intensity^36,37^; that is, VNS of moderate intensity (0.8 mA) can promote the plasticity of motor cortex and the recovery of upper limbs, while VNS of low-intensity (0.4 mA) and high-intensity (1.6 mA) have no significant promoting effect on the motor cortex.^36^ And reducing the current intensity and number of stimulations (Fast VNS) resulted in robust cortical plasticity.^37^ In addition, the augmentation of VNS current intensity can enhance thyroxine secretion in the cortex and hippocampus.^38^ However, it is important to note that more does not always equate to better outcomes, as current intensity increases, cortical plasticity decreases.^39^ Furthermore, other studies have also confirmed that VNS with higher current intensities and longer pulse widths drove greater increasing firing rate of cells in the locus coeruleus.^40^

Prolonging the treatment course from 4 to 8 weeks significantly improved the clinical efficacy of taVNS, highlighting the importance of treatment course. The sustained efficacy for 3 months further emphasized the benefits of the 8-week treatment. This was also what distinguished this study from previous research. A randomized clinical trial of electro-acupuncture treatment for insomnia combined with depression found that the best course of electro-acupuncture treatment for insomnia was 8 weeks, and the efficacy could be maintained for 6 months.^17^ Similarly, other studies have also found that only patients who received 8 weeks of taVNS had a significant decrease in office blood pressure and heart rate, further improvement in sympathetic balance, and a shift in circulating monocytes to an anti-inflammatory phenotype and endothelial cells to a reparative vascular profile.^41^

The study’s findings align with previous research indicating the relevance of the treatment course in achieving and maintaining therapeutic effects. However, determining the optimal treatment course for taVNS in patients with chronic insomnia disorder requires further investigation. In addition, the therapeutic effects of 8-week taVNS on clinical efficacy of chronic insomnia disorder was found to be sustained up to 3 months, which may be related to taVNS increasing the reactivity of insomnia (that is, improving the anxiety, depression, fatigue associated with insomnia) and regulating brain function (that is, reducing the excessive arousal of cerebral cortex). However, its long-term sustainability remains unexplored. Importantly, taVNS had good safety, tolerance, and compliance. Due to the advantages of simple and easy operation, taVNS is a promising nondrug therapy for insomnia. In the future, with the development of high-quality clinical studies on the treatment of insomnia, taVNS is expected to be an alternative therapy for CBT-I.

At present, there are relatively few studies on the mechanism of taVNS in treating insomnia. An animal study found that taVNS can significantly improve the power spectrum of δ band, reduce the power spectrum of β band in electroencephalogram (EEG) signals of insomnia model rats, and inhibit the excited state of cerebral cortex in insomnia model rats.^42^ Functional magnetic resonance imaging (MRI) studies have shown that taVNS can reduce the excitability of the cerebral cortex of patients with insomnia by modulating the nucleus tractus solitarius-limbus brain network and the functional connection in the default mode network.^7,14^ In future studies, we will use EEG and multimodal MRI techniques to further explore the mechanism of taVNS in the treatment of insomnia.

We also evaluated blinding to test whether patients knew the assignment at the end of treatment. The BBI results of sham taVNS group showed that the patients guessed in the opposite direction. The sham taVNS group believed that they were receiving taVNS treatment, so the sham taVNS group blind method was successful. This may have increased their treatment expectations to some extent, inevitably increasing the placebo effect. However, the clinical efficacy of the sham taVNS group was significantly reduced at the 20th week of follow-up, which is more likely to verify the success of the blind method in this group to a certain extent, and the actual clinical efficacy is weak.

### Limitations

This study has limitations. First, it used the maximum tolerable high intensity of stimulation, which does not fully confirm the effect of different stimulation intensities on the clinical efficacy of taVNS. Second, while taVNS was associated with improvements in sleep severity at week 4, these effects remained comparable to sham at week 20. This suggests that additional improvements may occur at later time points, warranting further studies to evaluate the long-term clinical efficacy of taVNS and to identify the optimal treatment regimen for chronic insomnia disorder. Additionally, participant recruitment in China was impacted by the COVID-19 pandemic, which may have influenced the severity of insomnia in the study population.

## Conclusions

This randomized clinical trial demonstrated the superiority of taVNS over sham control in treating chronic insomnia disorder. During the 8-week treatment period, taVNS significantly improved patient-reported outcomes, including sleep severity, fatigue, and mental health. Notably, clinically important reductions in PSQI were observed, with sustained effects comparable to sham at the end of 20 weeks. This study adds to the growing body of evidence supporting taVNS as a promising nonpharmacological intervention for chronic insomnia disorder.

## Notes

Supplement 1.
Trial Protocol

Supplement 2. **eFigure 1.** Illustration of taVNS and Sham taVNS **eFigure 2.** Forestplot of Blinding Assessment **eFigure 3.** Changes of PSQI Factor Scores in Two Groups **eFigure 4.** Change from Baseline in ISI, HAMD, HAMA, ESS, and FFS (Per-Protocol Set) **eTable 1.** Responses of Assessment of Blinding in the Trial **eTable 2.** Comparison of Primary and Secondary Outcomes in PP Set **eTable 3.** Difference of 7-Factor Score of PSQI Between the Group **eTable 4.** Adverse Events Related and Unrelated to Treatment

Supplement 3.
Data Sharing Statement
