# Supplementary material for: Transcutaneous Auricular Vagus Nerve Stimulation for Chronic Insomnia Disorder: A Randomized Clinical Trial
Source: JAMA Netw Open. 2024 Dec 16;7(12):e2451217. doi: 10.1001/jamanetworkopen.2024.51217 (PMC11650411; doi:10.1001/jamanetworkopen.2024.51217)
Supplement: Supplement 1. — Trial Protocol [file jamanetwopen-e2451217-s001.pdf]

STUDY PROTOCOL

Transcutaneous auricular vagus nerve stimulation for chronic insomnia disorder: a randomized clinical trial

Clinical sites:

Acupuncture Hospital of China Academy of Chinese Medical Sciences, Dongcheng District, Beijing, China

Confidentiality Statement

This document is the intellectual property of the Investigators. The information provided in this document is strictly confidential and is available for review to the sponsor, investigators, potential investigators, appropriate Ethics Committees, Investigational Review Boards, and other government regulatory bodies. No disclosure should take place without written authorization from the protocol developing investigators, except to the extent necessary needed to obtain informed consent from potential subjects.

STUDY CONTACT AND ORGANIZATION

Principal Investigator

Pei-Jing Rong, MD PhD

Institute of Basic Research in Clinical Medicine, China Academy of Chinese Medical Sciences, No. 16, Dongzhimen Nan Xiao Street, Dongcheng District, Beijing 100700, China.

Phone: +86 01064089301

Email: drrongpj@163.com

Co-Investigator for Recruiting

Acupuncture Hospital of China Academy of Chinese Medical Sciences

Ying Han, Chief Physician

E-mail: hanyingtcm@163.com

Acupuncture Hospital of China Academy of Chinese Medical Sciences

Shuai Zhang, PhD Candidate

E-mail: drzhangs187@126.com

Acupuncture Hospital of China Academy of Chinese Medical Sciences

Tuoran Wang, Attending physician

E-mail: 519827206@qq.com

Acupuncture Hospital of China Academy of Chinese Medical Sciences

Jin Huo, PhD Candidate

E-mail: hj20011998@163.com

INTRODUCTION

Chronic insomnia is a kind of sleep continuity disorder, accompanied by a series of daytime complaints such as sleepiness, fatigue, mood disorders or cognitive disorders, and sleep concerns and dissatisfaction with sleep,[1] which affects up to a third of the adult population worldwide.[2] Insomnia is the cause of many mental illnesses, and studies have shown that insomnia can increase the body's anxiety levels by 30% in the next day. Adequate, quality sleep can help people stay calm and reduce stress levels.[3] At present, the main treatment methods of insomnia are pharmacological therapy, cognitive behavioral therapy [4] and physical therapy, etc. Although pharmacotherapy and cognitive behavioral therapy for insomnia (CBT-I) therapy have made some advances, not all patients with insomnia respond to the recommended therapeutic options of CBT-I and pharmacological treatments.[1] Therefore, it is necessary to develop new alternative therapies.

As a non-pharmacological therapy, transcutaneous auricular vagus nerve stimulation (taVNS) has been applied in the treatment of depression,[5,6] epilepsy,[7,8] migraine[9] and other diseases. It can not only improve the insomnia symptoms of patients with depression,[5] but also play a great role in the treatment of insomnia.[10-13] A number of previous studies have shown that taVNS can not only effectively improve the sleep quality of insomnia patients, but also relieve their accompanying anxiety, depression, daytime sleepiness and fatigue symptoms[10,11,14]. Therefore, taVNS may offer a novel potential treatment modality for insomnia.

Stimulation parameters, such as frequency, intensity and treatment course, are the key factors affecting the clinical efficacy of taVNS. Previous studies have verified that different frequencies of taVNS can produce different physiological responses,[15-17] clinical efficacy[8,9] and brain effect mechanisms.[15,18] Different current intensities of taVNS also produce different physiological and clinical effects.[19-21] Although the initial clinical results of taVNS in the treatment of insomnia are encouraging, the stimulation parameters of the existing studies are relatively uniform, all of which are disperse-dense wave, 4/20Hz, and the stimulation intensity is mainly tolerated by patients without pain. The treatment course is 4 weeks, and the follow-up is 2 weeks to 6 weeks.[10,11,14] Therefore, the factors influencing the clinical efficacy of taVNS for insomnia remain unclear.

A number of previous studies have found an inverted-U response relationship between the efficacy of VNS and increased stimulus intensity.[22,23] However, there are relatively few studies on the effect of taVNS stimulus intensity on clinical efficacy, and the results of these studies are inconsistent[19,20,24]. Therefore, on the basis of verifying the efficacy and safety of taVNS in the treatment of insomnia,[10-13] this study further investigated whether the stimulus intensity of taVNS affected the efficacy of ID, and whether there is a difference in efficacy between high and low intensity stimulation.

HYPOTHESIS AND AIMS

The working hypothesis of the proposed study is that taVNS treatment for 8 weeks can significantly improve clinical outcomes in patients with insomnia, and the efficacy can be maintained for 3 months. To test this hypothesis, a 8-week, randomized controlled trial will be conducted in patients with insomnia to evaluate the efficacy of transcutaneous auricular vagus nerve stimulation (taVNS) on the clinical efficacy of insomnia patients, and to observe the 3-month efficacy of taVNS in the treatment of insomnia.

PLAN OF INVESTIGATION

Outline: A randomized controlled trial is designed to test the clinical efficacy of taVNS in the treatment of insomnia for 8 weeks and whether the efficacy can last for 3 months. To achieve this objective, 72 patients with insomnia will be recruited and randomly assigned to receive taVNS treatment (n =36) or sham taVNS treatment (n = 36) for 8 weeks and 12-weeks followed up. The primary outcome is Pittsburgh Sleep Quality Index (PSQI) score. Secondary and exploratory outcomes include PSQI components, response rate, Insomnia Severity Index (ISI), 17-item Hamilton Depression Scale (HAMD-17), 14-item Hamilton Anxiety Scale (HAMA-14), Epworth Sleepiness Scale (ESS), Flinders Fatigue Scale (FFS) and adverse events. The trial will be conducted in compliance with the protocol, Good Clinical Practice (GCP) and the applicable regulatory requirement(s).

Subjects

Diagnostic criteria: It meets the diagnostic criteria for insomnia disorder in the fifth edition of the Diagnostic and Statistical Manual of Mental Disorders (DSM-5).

1) Significant dissatisfaction with the quality or quantity of sleep, accompanied by at least one of the following symptoms:

①difficulty falling asleep;

②difficulty in sleep maintenance, characterized by frequent awakening or difficulty falling asleep after awakening;

③ Can't fall asleep again after waking up early.

2) Disturbed sleep (or associated daytime tiredness) causes significant distress or impairment in social, work, educational, academic, behavioral, or other important functions.

3) Sleeping difficulties occur at least 3 times a week.

4) Difficulty sleeping even when you get enough sleep opportunity.

5) Insomnia is not better explained by other sleep-wake disorders, nor does it occur during the course of other sleep-wake disorders (e.g., narcolepsy episodes, respiration-related sleep disorders, sleep-wake rhythm disorders, and parasomnia).

6) Insomnia is not caused by physical effects of substances (such as drug abuse).

7) Co-existing psychiatric and medical conditions are not sufficient to explain significant complaints of insomnia.

Inclusion criteria:

1) Meets the diagnostic criteria of DSM-5 insomnia disorder;

2) Insomnia symptoms lasting more than 3 months;

3) Chinese males and females aged 18 to 70 years;

4) Pittsburgh Sleep Quality Index (PSQI) score ≥8; 7≤HAMA score≤14, 7≤HAMD score≤17;

5) Didn't take any drugs or substances that act on the nervous system in the past month, and did not receive acupuncture treatment;

6) Can receive the treatment of auricular electrical stimulation;

7) Sign the informed consent.

Exclusion criteria: 1) Patients with severe physical diseases, such as coronary heart disease, malignant tumor, renal failure, etc.;

2) Patients with organic brain diseases;

3) with any other mental disorder, personality disorder or psychoactive substance abuse or dependence;

4) Patients who cannot be eluted by drugs or acupuncture;

5) Pregnant and lactating women;

6) Ear skin damage;

7) Unwilling to sign the informed consent form.

Estimation of sample size

This study was a prospective exploratory study. Using PSQI as the primary outcome and intervention and control group allocated as 1:1). Estimation of sample size is based on the results of the preliminary study,15 the mean PSQI score of patients with chronic insomnia is 14.9 (SD, 4.3), and the PSQI score is expected to be reduced to 11.1 after 8-weeks of taVNS treatment. Considering the dropout and loss to follow-up rate of approximately 25%, and with 0.05 α-level and 90% power, the total sample size required for the trial is 72 patients.

Screening, randomization and blindness

Eligible patients will be recruited from hospital outpatient clinics at Acupuncture and Moxibustion Hospital, China Academy of Chinese Medical Sciences (CACMS). If the recruitment could not reach the goal as the designed timetable frame at the end of the first year, additional study sites or other means of recruitment methods, such as advertisement, hospital WeChat official account and posters, will be considered to be included.

Screening will be conducted by the principal investigator following the inclusion and exclusion criteria. Randomization will be performed by an independent statistician in blocks with the use of SAS (version 9.4) software, with a block length of four. The randomized contents will be bound in closed envelopes,

and the order of each envelope is marked, which will be kept by a research assistant, but inaccessible to other study personnel. Remove envelopes according to the order in which the patients are enrolled.

After patient's eligibility is confirmed, patients will be randomly assigned to taVNS group or sham taVNS group in a ratio of 1:1 by informing as follows: "you will be randomly assigned to taVNS or sham taVNS treatment for your insomnia syndrome. You will have an equal chance to be assigned in either group, but either you or we will not know which group you will be assigned until investigational treatment completes." Clinical investigators, care providers, lab technicians, and data analysts will be blind to patients' treatment condition. The success of blinding will be tested by asking all participants the following question after the completion of the investigational treatment: "When you volunteered for the trial, you were informed that you had an equal chance of receiving taVNS or sham taVNS treatment. Which do you think you received?"

To assess the blinding maintenance of this study, the Bang's blinding index will be estimated. The primary purpose of BBI is to quantitatively evaluate the effectiveness of blinding. BBI assumes that in the absence of any unblinding, participants will guess their treatment group at random. For instance, in this two-arm trial (taVNS vs. sham taVNS), participants would have a 50% chance of guessing correctly purely by chance. The assumption also implies that each participant's guess is independent of others' guesses. This means that the blinding effectiveness is evaluated based on individual guesses without considering potential patterns or correlations between different participants' guesses. If the blinding is effective, the proportion of correct guesses should align closely with the expected proportion under random guessing. A higher than expected proportion of correct guesses suggests potential unblinding, meaning participants might have received cues or information that helped them correctly identify their treatment allocation. Conversely, a lower than expected proportion of correct guesses could indicate systematic bias or misinformation. As a statistical measure designed to assess the success of blinding in clinical trials, the estimation of BBI has been previously described in detail. In short, the BBI estimates the proportion of individuals who guess their treatment assignment correctly in the nth treatment arm. Each arm of a trial will therefore have its own BBI value, which is a continuous value and takes on a value between -1 and 1. If the BBI equals 1, it means that all responses are correct, and complete unblinding is inferred. If the BBI equals -1, then all responses are incorrect, and complete blinding is Inferred, although this may indicate unblinding in the opposite direction (e.g. opposite guessing). If the BBI equals 0, then half of the guesses are correct and half of the guesses are incorrect, inferring random guessing. In general, if the BBI takes on value from -0.2 to 0.2, blinding is considered to be successful. Unblinding (or opposite guessing) may be claimed if the relevant limit of the 95% confidence interval does not cover 0.

Unblinding Procedures

The study treatment blind shall not be broken by the investigator unless information concerning the

study treatment is necessary for the medical treatment of the subject. In the event of a medical emergency, if possible, the medical monitor should be contacted before the study treatment blind is broken to discuss the need for unblinding. For unblinding a subject, the study treatment blind can be obtained by the principal investigator. If any site personnel are unblinded, the dosing of the study treatment would be stopped immediately and the subject would be withdrawn from the study. The date, time, and reason the blind is broken would be recorded.

Treatment procedure

The intervention protocol was based on a preliminary clinical trial [10], utilized an electroacupuncture instrument (Hwato, SDZ-II B, Suzhou, China) and special silica gel ear clips. Patients in both groups received electrical stimulation at the bilateral auricular cymba conchae(auricular point kidney, CO_{10}) and cavum conchae(auricular point heart, CO_{15}), where there is rich of vagus nerve distribution [25]. Patients in taVNS group received stimulation parameters, including dilatational wave at 4/20Hz (4Hz for 5 seconds, 20Hz for 10 seconds, alternately), pulse width of 0.2ms±30%, and intensity adjusted to the patient's maximum tolerable level (0.8~1.5mA). The sham taVNS group received the same parameters with a current intensity of 0.1mA.

On the day of enrollment, each patient will be taught to use the instrument, informed of precautions, and ensure that each patient could treat himself at home. Patients in both groups starte treatment on the day of randomization and bilateral auricles receive stimulation simultaneously for 30 min each time, twice a day, five consecutive days a week, for 8 weeks, followed by a 12-week observational period. Sleep treatment diary will be used to monitor the patient's treatment and sleep status.

Patients are encouraged to avoid using medications or other therapies for treatment of insomnia throughout the trial. In accordance with the Declaration of Helsinki, if patients have severe insomnia and affect their daily life, temporary use of sleeping pills is allowed (the frequency of continuous use of drugs is allowed to be less than 3 nights), and the name and dose of drugs are recorded in the sleep diary and informed to the responsible doctor. For those who were under anti-hyperlipidemic, antihypertensive and anti-diabetic treatment, they were allowed to continue their current medications throughout the study. Drug history of the participant will be systematically recorded in the case report form.

Monitoring of treatment compliance

Self-report daily diary will be used to monitor treatment compliance. The treatment diary will record the number of times and the length of the treatment each day. The compliance is calculated by taking the number of times the patient is actually treated divided by the number of times the patient should be treated and multiply by 100.

Clinical and biochemical assessment

Efficacy: The efficacy of the taVNS for chronic insomnia disorder will be associated with the primary outcome and secondary outcome.

The primary outcome is PSQI. The primary endpoint is the mean changes from baseline through week 8 in PSQI scores.

Secondary outcomes include the mean changes in PSQI scores at 4,20 weeks, the response rate at 4, 8 and 20 weeks, as well as the changes in seven components of PSQI, Insomnia Severity Index (ISI), Hamilton Depression Scale (HAMD-17), Hamilton Anxiety Scale (HAMA-14), Epworth Sleepiness Scale (ESS), and Flinders Fatigue Scale (FFS) from baseline to 4, 8 and 20 weeks. Adherence is monitored through the sleep treatment diary. Additionally, adverse reactions are recorded. The response rate was defined as the percentage of patients with a reduction of ≥50% from baseline in the PSQI score after treatment.

Other clinical assessments will be conducted at baseline, 4 weeks,8weeks and 20 weeks. Blood pressure will be measured by clinical sphygmomanometer with the cuff placed around the upper arm. It will be measured in the morning at resting state after sitting quietly for at least 15 minutes. The measurement will be conducted in the same arm and no talking will be allowed during the measurement.

Safety: The safety of the taVNS will be associated with adverse events (AEs) reported by participants and recorded by physician. The incidence, intensity, subjective feeling of the relationship with the treatment, and management of the event will be recorded. Safety assessment will be conducted at 4 weeks, and 8 weeks. The reasons for withdrawal will be recorded when a subject withdraws before completing the study. Serious AEs are defined as any undesirable experience that is fatal, is life-threatening, requires initial or prolonged in-patient hospitalization, or causes persistent or significant disability or permanent damage.

Data analyses

The analysis will be performed on the intention-to-treat (ITT) population that participants had been randomized.All efficacy analyses will be performed using the ITT analysis set and PP analysis set, defined as all randomized participants who received at least 6-week intervention. A mixed model for repeated measures (MMRM) with likelihood-based modeling to handle missing data will be applied to compare the primary outcome and secondary outcomes: change in the PSQI, ISI, HAMD, HAMA, ESS, FFS scores from baseline to week 4, 8, and 20. The model includes terms for treatment (taVNS or sham taVNS), visit (weeks 4, 8, 20), treatment by visit interaction, and baseline scores. Least squares mean change from baseline and least square mean difference between the groups at week 8 along with 95% confidence interval and a 2-sided p value will be estimated for the primary end point. Cohen d effect size will be calculated using the absolute value of the least square mean difference between groups in score from baseline at week 8 divided by the pooled SD estimated from the mixed model for repeated measures. For the PSQI responder analysis, the responder is defined as a 50% decrease at the end of treatment compared with baseline, which is prespecified in the statistical analysis plan. The percentage of PSQI responder at week 4, 8 and 20 will be calculated and summarized by treatment group. The percentage of PSQI responders at week 8 and 20 will be compared between groups using the Chi-square test. SAS, version 9.4 (SAS Institute Inc) is used for all analyses, with significance set at $P < 0.05$.

Premature Termination or Suspension of Study

The study will be completed as planned unless one or more of the following criteria are satisfied that require temporary suspension or early termination of the study: (1) Patients who developed severe adverse reactions were unable to continue with the scheduled experimenter; (2) significant violation of GCP that compromises the ability to achieve the primary study objectives or compromises subject safety.

Procedures for Premature Termination or Suspension

In the event that the principal investigator, an institutional review board (IRB) / research ethics committee (REC) or regulatory authority elects to terminate or suspend the study or the participation of an investigational site, a study-specific procedure for early termination or suspension will be provided by the principal investigator; the procedure will be followed by applicable investigational sites during the course of termination or study suspension.

Criteria for Discontinuation or Withdrawal of a Subject

(1) *Pretreatment event or AE*: The subject has experienced a pretreatment event or AE that requires early termination because continued participation imposes an unacceptable risk to the subject's health or the subject is unwilling to continue because of the pretreatment event or AE. (2) *Major protocol deviation*: The discovery post-randomization that the subject failed to meet protocol entry criteria or

did not adhere to protocol requirements, and continued participation poses an unacceptable risk to the subject's health. (3) *Lost to follow-u*p: The subject did not return to the hospital and attempts to contact the subject were unsuccessful. (4) *Withdrawal of consent*: The subject wishes to withdraw from the study. (5) *Study termination*: The principal investigator, IRB, REC, or regulatory agency terminates the study.

Procedures for Discontinuation or Withdrawal of a Subject

The investigator may terminate a subject's study participation at any time during the study when the subject meets the study termination criteria. In addition, a subject may discontinue his or her participation without giving a reason at any time during the study. Should a subject's participation be discontinued, the primary criterion for termination would be recorded. Subjects who discontinue the study during the double-blind treatment period will visit the site as soon as possible for clinical assessment if applicable. Discontinued or withdrawn subjects will not be replaced.

Record retention

The principal investigator, research team, IRB / REC are responsible for overseeing this study to get access to, to use, and to retain subjects' personal data for the purposes of clinical study; and the relevant regulator bodies can get access to subjects' personal data for the purposes of checking and verifying the integrity of study data and assessing compliance with the study protocol and relevant requirements. The data are expected to be stored for at least 3 years, and be destroyed after that, subject to local regulations.

Publication policy

The results of this study are going to be published in peer-review journals, with abstract available online at clinical trial register. Subjects enjoy rights for the protection of the confidentiality of their personal data.

Anticipated results

The most expected and scientifically significant findings should be that taVNS treatment may achieved clinically significant improvements, without aggravating insomnia symptoms and other adverse events, compared to sham taVNS.

PURPOSE AND POTENTIAL

Chronic insomnia disorder is a common disorder. At present, the main treatment methods are drugs and cognitive behavior therapy, but there are certain limitations. Transcutaneous auricular vagus nerve stimulation is a non-pharmacological intervention, has shown efficacy in treating insomnia. However, the factors that affect the clinical curative effect is unclear. If the results obtained in the proposed study is positive, it will provide direct evidence to support the use of taVNS as an effective therapy for

chronic insomnia disorder.

ETHICAL CONSIDERATION

Ethical approval from the ethics committee of the Institute of Acupuncture and Moxibustion, CACMS. The research protocol will comply with the Declaration of Helsinki and its subsequent amendment and be filed for approval by IRB. The trial will be registered in the Chinese Clinical Trial Registry, which is accessible to the public, before the onset of patient enrollment. A full explanation of the study goal, procedures, and potential side effects and risks will be presented to each patient recruited. Patients must provide a written, informed consent for participating into the study. Patients would not receive remuneration, but treatments and sleep psychological scale evaluations directly associated with the clinical trial will be provided at no costs. This study is conducted in Chinese patients. The issue of confidentiality is the major ethical issue, and will be solved by recording the data in a manner that does not allow the participants to be identified (ie. using a non-recognizable code for each patient). A review of medical records that have been already recorded as part of clinical care, therefore this poses no physical risks.

REFERENCES

1. Perlis ML, Posner D, Riemann D, Bastien CH, Teel J, Thase M. Insomnia. Lancet (London, England). 2022;400(10357):1047-1060.
2. Perlis ML, Pigeon WR, Grandner MA, et al. Why Treat Insomnia? Journal of primary care & community health. 2021;12:21501327211014084.
3. Ben Simon E, Rossi A, Harvey AG, Walker MP. Overanxious and underslept. Nature human behaviour. 2020;4(1):100-110.
4. Soong C, Burry L, Greco M, Tannenbaum C. Advise non-pharmacological therapy as first line treatment for chronic insomnia. BMJ (Clinical research ed). 2021;372:n680.
5. Fang J, Rong P, Hong Y, et al. Transcutaneous Vagus Nerve Stimulation Modulates Default Mode Network in Major Depressive Disorder. Biological psychiatry. 2016;79(4):266-273.
6. Li S, Rong P, Wang Y, et al. Comparative Effectiveness of Transcutaneous Auricular Vagus Nerve Stimulation vs Citalopram for Major Depressive Disorder: A Randomized Trial. Neuromodulation : journal of the International Neuromodulation Society. 2022;25(3):450-460.
7. Rong P, Liu A, Zhang J, et al. An alternative therapy for drug-resistant epilepsy: transcutaneous auricular vagus nerve stimulation. Chinese medical journal. 2014;127(2):300-304.
8. Bauer S, Baier H, Baumgartner C, et al. Transcutaneous Vagus Nerve Stimulation (tVNS) for Treatment of Drug-Resistant Epilepsy: A Randomized, Double-Blind Clinical Trial (cMPsE02). Brain stimulation. 2016;9(3):356-363.
9. Straube A, Ellrich J, Eren O, Blum B, Ruscheweyh R. Treatment of chronic migraine with transcutaneous stimulation of the auricular branch of the vagal nerve (auricular t-VNS): a randomized, monocentric clinical trial. The journal of headache and pain. 2015;16:543.

10. Jiao Y, Guo X, Luo M, et al. Effect of Transcutaneous Vagus Nerve Stimulation at Auricular Concha for Insomnia: A Randomized Clinical Trial. Evidence-based complementary and alternative medicine : eCAM. 2020;2020:6049891.

11. Wu Y, Song L, Wang X, et al. Transcutaneous Vagus Nerve Stimulation Could Improve the Effective Rate on the Quality of Sleep in the Treatment of Primary Insomnia: A Randomized Control Trial. Brain sciences. 2022;12(10).

12. Zhang S, He JK, Meng H, et al. Effects of transcutaneous auricular vagus nerve stimulation on brain functional connectivity of medial prefrontal cortex in patients with primary insomnia. Anatomical record (Hoboken, NJ : 2007). 2021;304(11):2426-2435.

13. Wu X, Zhang Y, Luo WT, et al. Brain Functional Mechanisms Determining the Efficacy of Transcutaneous Auricular Vagus Nerve Stimulation in Primary Insomnia. Frontiers in neuroscience. 2021;15:609640.

14. Luo M, Qu X, Li S, et al. [Transcutaneous vagus nerve stimulation for primary insomnia and affective disorder:a report of 35 cases]. Zhongguo zhen jiu = Chinese acupuncture & moxibustion. 2017;37(3):269-273.

15. Sclocco R, Garcia RG, Kettner NW, et al. Stimulus frequency modulates brainstem response to respiratory-gated transcutaneous auricular vagus nerve stimulation. Brain stimulation. 2020;13(4):970-978.

16. Badran BW, Mithoefer OJ, Summer CE, et al. Short trains of transcutaneous auricular vagus nerve stimulation (taVNS) have parameter-specific effects on heart rate. Brain stimulation. 2018;11(4):699-708.

17. Steidel K, Krause K, Menzler K, et al. Transcutaneous auricular vagus nerve stimulation influences gastric motility: A randomized, double-blind trial in healthy individuals. Brain stimulation. 2021;14(5):1126-1132.

18. Cao J, Zhang Y, Li H, et al. Different modulation effects of 1 Hz and 20 Hz transcutaneous auricular vagus nerve stimulation on the functional connectivity of the periaqueductal gray in patients with migraine. J Transl Med. 2021;19(1):354.

19. Šinkovec M, Trobec R, Kamenski T, Jerman N, Meglič B. Hemodynamic responses to low-level transcutaneous auricular nerve stimulation in young volunteers. IBRO neuroscience reports. 2023;14:154-159.

20. Capone F, Motolese F, Di Zazzo A, et al. The effects of transcutaneous auricular vagal nerve stimulation on pupil size. Clinical neurophysiology : official journal of the International Federation of Clinical Neurophysiology. 2021;132(8):1859-1865.

21. Sclocco R, Garcia RG, Gabriel A, Kettner NW, Napadow V, Barbieri R. Respiratory-gated Auricular Vagal Afferent Nerve Stimulation (RAVANS) effects on autonomic outflow in hypertension. Annual International Conference of the IEEE Engineering in Medicine and Biology Society IEEE Engineering in Medicine and Biology Society Annual International Conference. 2017;2017:3130-3133.

22. Borland MS, Vrana WA, Moreno NA, et al. Cortical Map Plasticity as a Function of Vagus Nerve

Stimulation Intensity. Brain stimulation. 2016;9(1):117-123.

23. Loerwald KW, Borland MS, Rennaker RL, 2nd, Hays SA, Kilgard MP. The interaction of pulse width and current intensity on the extent of cortical plasticity evoked by vagus nerve stimulation. Brain stimulation. 2018;11(2):271-277.

24. Borges U, Laborde S, Raab M. Influence of transcutaneous vagus nerve stimulation on cardiac vagal activity: Not different from sham stimulation and no effect of stimulation intensity. PloS one. 2019;14(10):e0223848.

25. Peuker ET, Filler TJ. The nerve supply of the human auricle. Clinical anatomy (New York, NY). 2002;15(1):35-37.
