# Supplementary material for: Transcutaneous Auricular Vagus Nerve Stimulation for Chronic Insomnia Disorder: A Randomized Clinical Trial
Source: JAMA Netw Open. 2024 Dec 16;7(12):e2451217. doi: 10.1001/jamanetworkopen.2024.51217 (PMC11650411; doi:10.1001/jamanetworkopen.2024.51217)
Supplement: Supplement 2. — eFigure 1. Illustration of taVNS and Sham taVNS eFigure 2. Forestplot of Blinding Assessment eFigure 3. Changes of PSQI Factor Scores in Two Groups eFigure 4. Change from Baseline in ISI, HAMD, HAMA, ESS, and FFS (Per-Protocol Set) eTable 1. Responses of Assessment of Blinding in the Trial eTable 2. Comparison of Primary and Secondary Outcomes in PP Set eTable 3. Difference of 7-Factor Score of PSQI Between the Group eTable 4. Adverse Events Related and Unrelated to Treatment [file jamanetwopen-e2451217-s002.pdf]

Supplemental Online Content

Zhang S, Zhao Y, Qin Z, et al. Transcutaneous auricular vagus nerve stimulation for chronic insomnia disorder: a randomized clinical trial. *JAMA Netw Open.* 2024;7(12):e2451217. doi:10.1001/jamanetworkopen.2024.51217

eFigure 1. Illustration of taVNS and Sham taVNS

eFigure 2. Forestplot of Blinding Assessment

eFigure 3. Changes of PSQI Factor Scores in Two Groups

eFigure 4. Change from Baseline in ISI, HAMD, HAMA, ESS, and FFS (Per-Protocol Set)

eTable 1. Responses of Assessment of Blinding in the Trial

eTable 2. Comparison of Primary and Secondary Outcomes in PP Set

eTable 3. Difference of 7-Factor Score of PSQI Between the Group

eTable 4. Adverse Events Related and Unrelated to Treatment

This supplemental material has been provided by the authors to give readers additional information about their work.

© 2024 Zhang S et al. *JAMA Network Open.*

SUPPLEMENTARY FIGURES

eFigure 1. Illustration of taVNS and sham taVNS

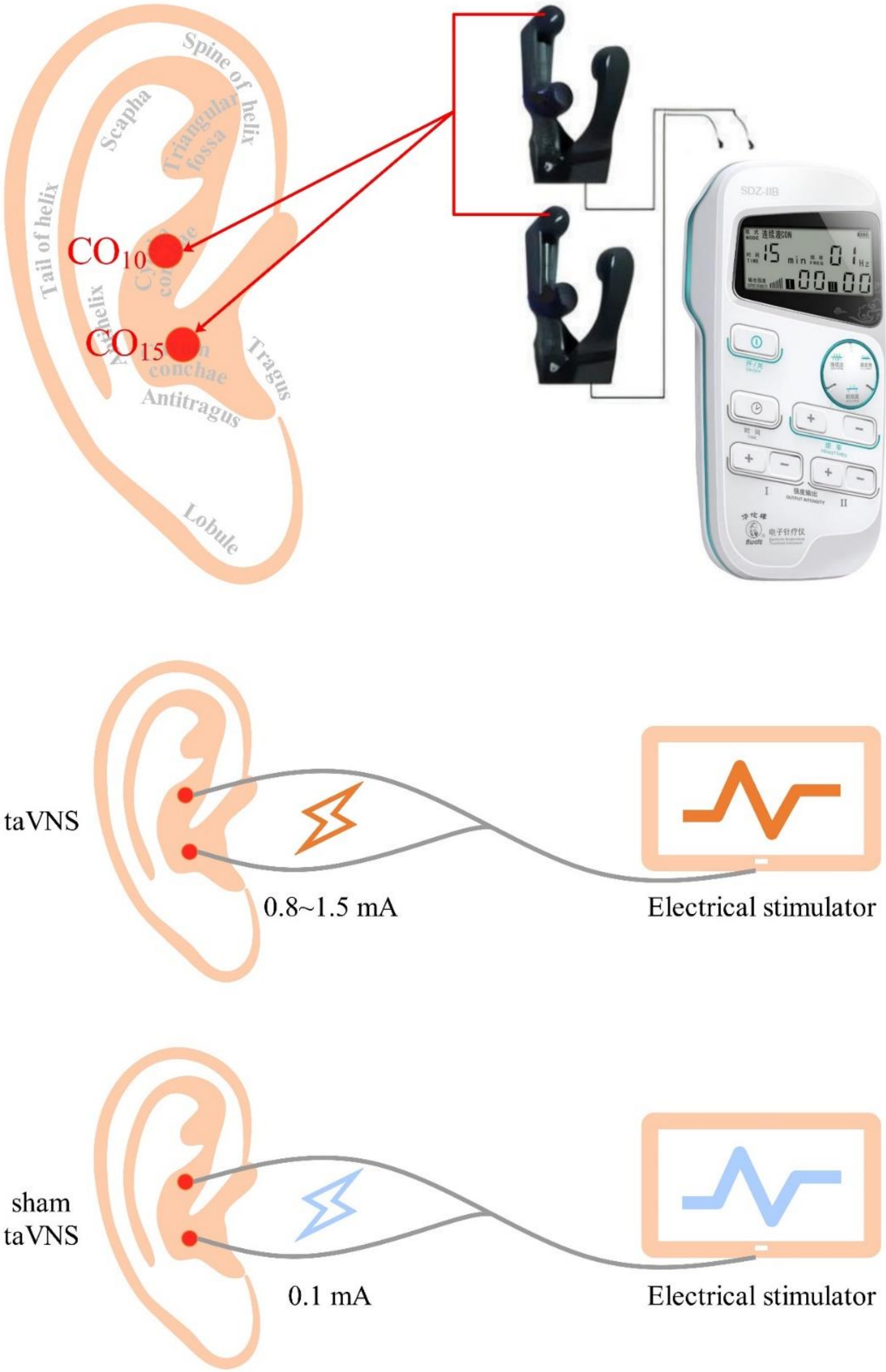

Abbreviations: taVNS. Transcutaneous auricular vagus nerve stimulation.

© 2024 Zhang S et al. *JAMA Network Open.*

eFigure 2. Forestplot of Blinding Assessment

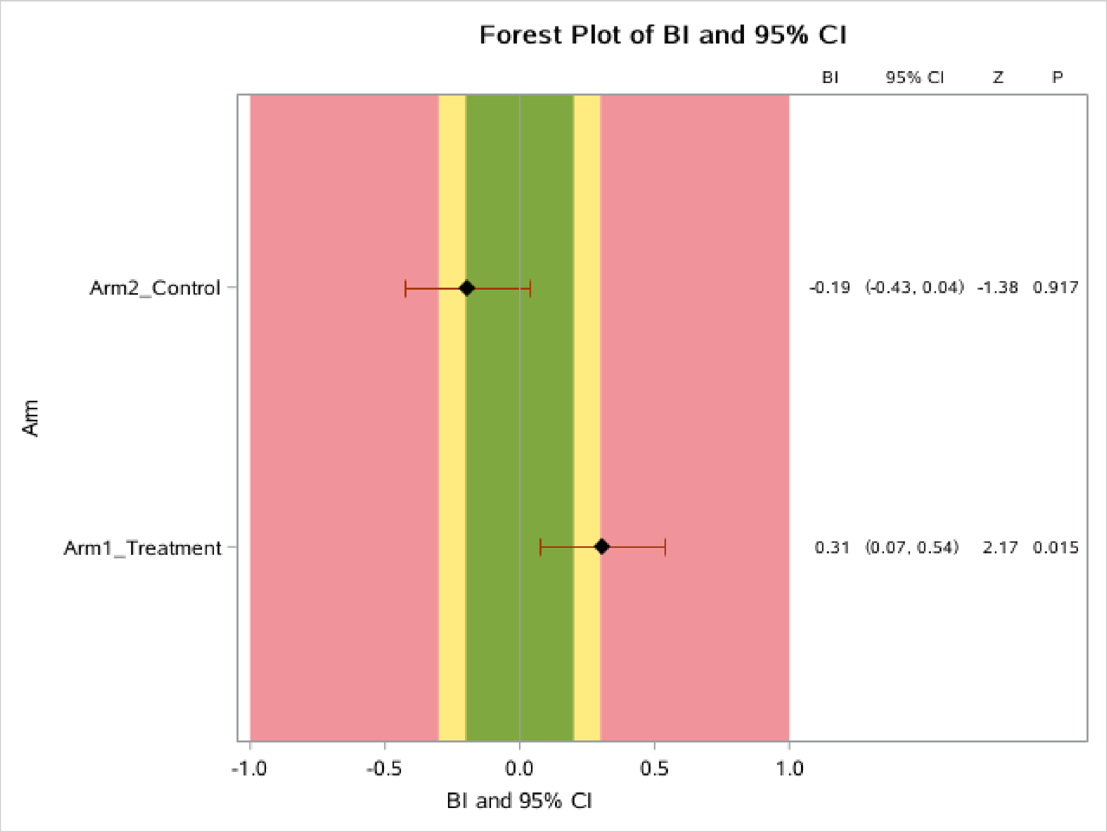

Note: Arm1_Treatment for taVNS vs. Arm2_Control for sham taVNS.

Abbreviations: BI, Bang's blinding index; CI, confidence interval

© 2024 Zhang S et al. *JAMA Network Open.*

eFigure 3. Changes of PSQI Factor Scores in two groups

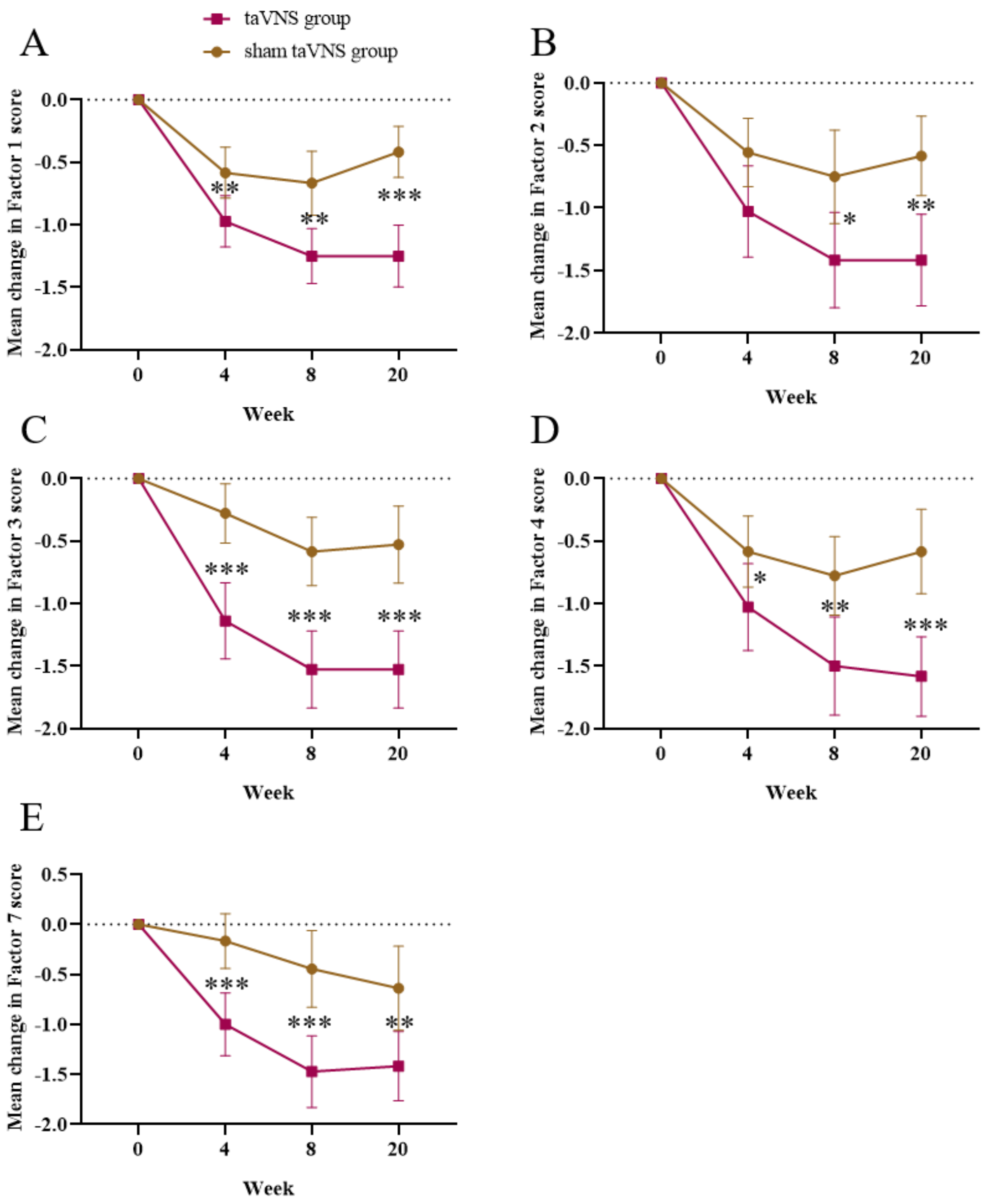

Note: A, Comparison of changes in subjective sleep quality scores between the two groups; B, Comparison of changes in sleep latency scores between the two groups; C, Comparison of changes in sleep duration scores between the two groups; D, Comparison of changes in sleep efficiency scores between the two groups; E, Comparison of changes in daytime dysfunction scores between the two groups. ***P<0.001, **P<0.01, *P<0.05. Abbreviations: taVNS. Transcutaneous auricular vagus nerve stimulation.

© 2024 Zhang S et al. *JAMA Network Open.*

eFigure 4. Change from baseline in ISI, HAMD, HAMA, ESS, and FFS (Per-Protocol Set)

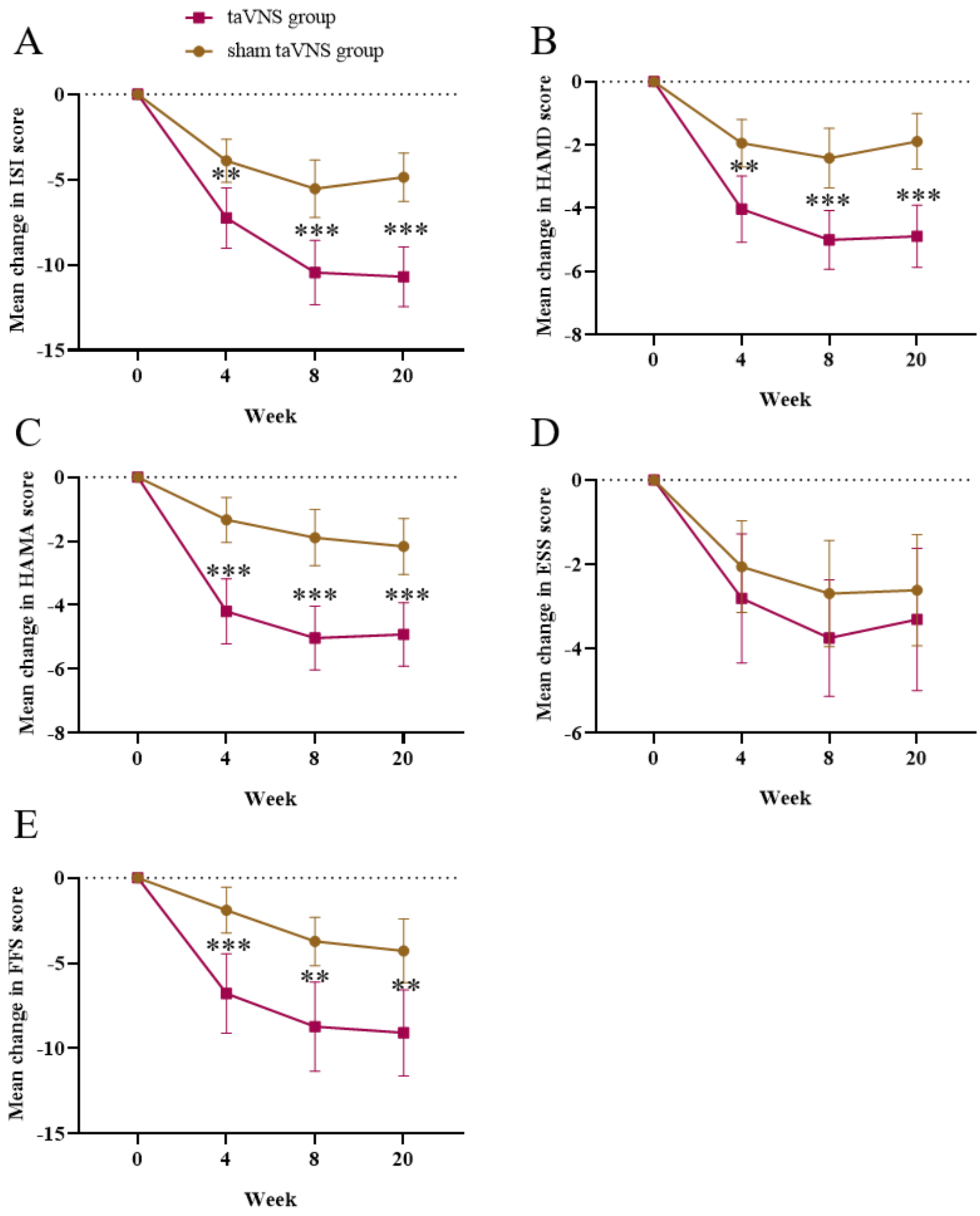

Note: A, Comparison of mean changes in ISI score between the two groups; B, Comparison of mean changes in HAMD score between the two groups; C, Comparison of mean changes in HAMA score between the two groups; D, Comparison of mean changes in ESS score between the two groups; E, Comparison of mean changes in FFS score between the two groups. ****P*<0.001, ***P*<0.01.

Abbreviations: taVNS. Transcutaneous auricular vagus nerve stimulation.

© 2024 Zhang S et al. *JAMA Network Open.*

SUPPLEMENTARY TABLES

eTable 1. Responses of assessment of blinding in the trial

Guessed, n (%)	True arm	
	TaVNS	Sham taVNS
**TaVNS**	20 (55.6)	17 (47.2)
**Sham taVNS**	9 (25.0)	10 (27.8)
**Unknown**	7 (19.4)	9 (25.0)

Abbreviations: taVNS. Transcutaneous auricular vagus nerve stimulation.

© 2024 Zhang S et al. *JAMA Network Open.*

eTable 2. Comparison of primary and secondary outcomes in PP set

Outcome	taVNS (*n*=35)	Sham taVNS (*n*=33)	*P* value	Difference(95%CI)
Primary outcome				
**Mean change in PSQI score (95% CI)**				
Week 4	-6.0 (-7.2 to -4.9)	-2.6 (-3.6 to -1.7)	< .001	-3.4 (-4.9 to -1.9)
Week 8	-8.2 (-9.4 to -7.0)	-3.9 (-5.2 to -2.6)	< .001	-4.0 (-6.0 to -3.0)
Week 20	-8.0 (-9.2 to -6.9)	-3.4 (-4.8 to -2.0)	< .001	-4.6 (-6.4 to -2.8)
Secondary outcomes				
**Responders (95% CI), %**				
Week 4	51.4 (34.0 to 68.8)	18.2 (4.3 to 32.1)	0.004	33.2 (12 to 54.4)
Week 8	71.4 (55.7 to 87.2)	33.3 (16.4 to 50.3)	0.002	38.1 (16.1 to 60.1)
Week 20	74.3 (59.1 to 89.5)	24.2 (8.8 to 39.7)	< .001	50.1 (29.5 to 70.7)
**Mean change in ISI score (95% CI)**				
Week 4	-7.5(-9.2 to -5.7)	-4.1 (-5.5 to -2.8)	0.003	-3.3 (-5.5 to -1.1)
Week 8	-10.7(-12.6 to -8.9)	-5.9 (-7.7 to -4.1)	< .001	-4.8 (-7.4 to -2.3)
Week 20	-11.0(-12.7 to -9.3)	-5.2 (-6.7 to -3.7)	< .001	-5.8 (-8.0 to -3.6)
**Mean change in HAMD score (95% CI)**				
Week 4	-4.1 (-5.2 to -3.1)	-2.0 (-2.8 to -1.2)	0.002	-2.1 (-3.4 to -0.8)
Week 8	-5.1 (-6.1 to -4.2)	-2.6 (-3.6 to -1.5)	< .001	-2.6 (-3.9 to -1.3)
Week 20	-5.0 (-6.0 to -4.1)	-2.0 (-2.9 to -1.0)	< .001	-3.1 (-4.4 to -1.7)
**Mean change in HAMA score (95% CI)**				
Week 4	-4.3 (-5.3 to -3.3)	-1.3 (-2.1 to -0.6)	< .001	-3.0 (-4.0 to -2.0)
Week 8	-5.2 (-6.2 to -4.2)	-1.9 (-2.9 to -1.0)	< .001	-3.0 (-5.0 to -2.0)
Week 20	-5.1 (-6.0 to -4.1)	-2.2 (-3.2 to -1.3)	< .001	-2.8 (-4.1 to -1.5)
**Mean change in ESS score (95% CI)**				
Week 4	-2.9 (-4.5 to -1.3)	-2.1 (-3.3 to -0.9)	0.670	0 (-2.0 to 1.0)
Week 8	-3.9 (-5.3 to -2.5)	-2.8 (-4.2 to -1.4)	0.270	-1.1 (-2.9 to 0.9)
Week 20	-3.4 (-5.1 to -1.7)	-2.7 (-4.1 to -1.3)	0.528	-0.7 (-2.9 to 1.5)
**Mean change in FFS score (95% CI)**				
Week 4	-7.0 (-9.3 to -4.6)	-2.0 (-3.5 to -0.6)	0.001	-4.9 (-7.7 to -2.2)
Week 8	-9.0 (-11.6 to -6.3)	-4.0 (-5.5 to -2.5)	0.002	-4.9 (-7.9 to -1.9)
Week 20	-9.3 (-11.9 to -6.8)	-4.6 (-6.6 to -2.6)	0.005	-4.7 (-7.9 to -1.5)

Abbreviation: taVNS. Transcutaneous auricular vagus nerve stimulation; PSQI, Pittsburgh Sleep Quality Index; ISI, Insomnia severity index; HAMD-17, 17-item Hamilton depression; HAMA-14, 14-item Hamilton anxiety; ESS, Epworth sleepiness; FFS, Flinders fatigue.

© 2024 Zhang S et al. *JAMA Network Open.*

eTable 3. Difference of 7-factor score of PSQI between the group

Outcome	taVNS (*n*=36)	Sham taVNS (*n*=36)	*P* value
**Factor 1**			
Week 4	-1.0 (-1, -1)	-1.0 (-1, 0)	0.008
Week 8	-1.0 (-2, -1)	-1.0 (-1, 0)	0.001
Week 20	-1.0 (-2, -1)	0 (-1, 0)	＜.001
**Factor 2**			
Week 4	-1.0 (-2, 0)	0 (-1, 0)	0.072
Week 8	-2.0 (-2, 0)	-1.0 (-2, 0)	0.019
Week 20	-1.0 (-2, -0.5)	0 (-1, 0)	0.001
**Factor 3**			
Week 4	-1.0 (-2, -0.5)	0 (-1, 0)	< .001
Week 8	-2.0 (-2, -1)	-1.0 (-1, 0)	< .001
Week 20	-2.0 (-2, -1)	0 (-1, 0)	< .001
**Factor 4**			
Week 4	-1.0 (-2, 0)	0 (-1, 0)	0.048
Week 8	-1.0 (-3, -1)	-1.0 (-1, 0)	0.006
Week 20	-2.0 (-2, -1)	0 (-1, 0)	< .001
**Factor 5**			
Week 4	-1.0 (-1, 0)	-1.0 (-1, 0)	0.131
Week 8	-1.0 (-1, 0)	0 (-1, 0)	0.270
Week 20	0 (-1, 0)	-1.0 (-1, 0)	0.639
**Factor 6**			
Week 4	0 (0, 0)	0 (0, 0)	0.585
Week 8	0 (0, 0)	0 (0, 0)	0.286
Week 20	0 (0, 0)	0 (0, 0)	0.179
**Factor 7**			
Week 4	-1.0 (-2, 0)	0 (-1, 0)	< .001
Week 8	-2.0 (-2, -1)	0 (-1, 0)	< .001
Week 20	1.0 (-2, -1)	0 (-1.5, 0)	0.004

Note: The data were represented as median and interquartile ranges; Factor 1, subjective sleep quality; Factor 2, sleep latency; Factor 3, sleep duration; Factor 4, habitual sleep efficiency; Factor 5, sleep disturbance; Factor 6, use of hypnotic drugs; Factor 7, daytime dysfunction.

Abbreviations: taVNS, Transcutaneous auricular vagus nerve stimulation.

© 2024 Zhang S et al. *JAMA Network Open.*

eTable 4. Adverse Events Related and Unrelated to Treatment

Adverse Event	taVNS (*n*=36)	Sham taVNS (*n*=36)	*P* value
Any	5 (13.9 %)	4 (11.1%)	1.000
Related to treatment			
Slight clamping pain in the outer ear	4 (11.1%)	3 (8.3%)	
Unrelated to treatment			
Bleeding gums	1 (2.8%)	0	
Vertigo	0	1 (2.8%)	
Serious	0	0	

Note: Descriptive statistics were used for adverse reactions. *P* value is the result of the Fisher exact test.

Abbreviations: taVNS, Transcutaneous auricular vagus nerve stimulation.

© 2024 Zhang S et al. *JAMA Network Open.*
