# Supplementary material for: Transcutaneous Auricular Vagus Nerve Stimulation for Chronic Insomnia Disorder: A Randomized Clinical Trial
Source: JAMA Netw Open. 2024 Dec 16;7(12):e2451217. doi: 10.1001/jamanetworkopen.2024.51217 (PMC11650411; doi:10.1001/jamanetworkopen.2024.51217)
Supplement: Supplement 3. — Data Sharing Statement [file jamanetwopen-e2451217-s003.pdf]

Data Sharing Statement

Zhang. Transcutaneous Auricular Vagus Nerve Stimulation for Chronic Insomnia Disorder. *JAMA Netw Open*. Published December 16, 2024. doi:10.1001/jamanetworkopen.2024.51217

Data
Additional Information: Chinese Clinical Trial Registry: ChiCTR2100051319
Data available: Yes
Data types: Data dictionary
How to access data: No data will be shared prior to publication of the results of the study in the Journal and in the report to the funder. Applications should be emailed to the Co-Investigator, Yu Wang, at wy8166@126.com
When available: beginning date: 12-31-2024

Supporting Documents
Document types: None

Additional Information
Who can access the data: We will be open to applications for collaborations with other researchers to use the study data after the publication of this study, as our research ethics permission is for the use of the data by the research team only.
Types of analyses: For any purpose
Mechanisms of data availability: Applications to use the data should provide a peer-reviewed study protocol. The decision will be considered by the 3 investigators (Dr. Zongshi Qin, Dr. Yu Wang, and Dr. Peijing Rong) on data sharing.
